# Genetic Diversity and Epidemiology of Enteroviruses and Rhinoviruses in Children Hospitalized with Acute Respiratory Infections in Novosibirsk, Russia (2023–2024)

**DOI:** 10.3390/v16121924

**Published:** 2024-12-16

**Authors:** Alina R. Nokhova, Tereza A. Saroyan, Mariya V. Solomatina, Tatyana A. Gutova, Anastasiya A. Derko, Nikita A. Dubovitskiy, Tatyana A. Murashkina, Kirill A. Sharshov, Alexander M. Shestopalov, Olga G. Kurskaya

**Affiliations:** Federal Research Center of Fundamental and Translational Medicine, Novosibirsk 630060, Russia; alina.nokhova@gmail.com (A.R.N.); 111.st.13@rambler.ru (T.A.S.); solomatina.mariyav@yandex.ru (M.V.S.); a.derko19@gmail.com (A.A.D.); nikitadubovitskiy@gmail.com (N.A.D.); tamurashkina@frcftm.ru (T.A.M.); sharshov@yandex.ru (K.A.S.); shestopalov2@mail.ru (A.M.S.)

**Keywords:** enteroviruses, rhinoviruses, *Picornaviridae*, EV typing, EV-C105, EV-D68

## Abstract

Rhinoviruses and respiratory enteroviruses remain among the leading causes of acute respiratory infections, particularly in children. Little is known about the genetic diversity of enteroviruses and rhinoviruses in pediatric patients with acute respiratory infections in Russia. We assessed the prevalence of human rhinoviruses/enteroviruses (HRV/EV) in 1992 children aged 0 to 17 years hospitalized with acute respiratory infections during the 2023–2024 epidemic season using PCR. The detection rate of HRV/EV was 11% (220/1992). We performed typing of 58 HRV and 28 EV viruses by partial sequencing of the VP1 gene. Rhinovirus A was the most common among HRV, followed by rhinovirus C; rhinovirus B was detected in only three cases. Enteroviruses were represented by all four species, with the EV-D68 genotype being the most frequently detected. Phylogenetic analysis of the VP1 fragment of EV-D68 showed that all our sequences belonged to the B3 subclade. We identified the first case of EV-C105 infection in Russia in a two-year-old girl hospitalized with pneumonia. Phylogenetically, the Novosibirsk strain EV-C105 was closely related to a strain discovered in France in 2018. This research helped to fill a critical gap in understanding the epidemiological landscape of HRV/EV in pediatric populations within Russia.

## 1. Introduction

Enteroviruses (EV) and Human Rhinoviruses (HRV) belong to the genus Enterovirus of the Picornaviridae family, which is classified into 15 species (EV A–L and HRV A–C). EV A–D and HRV A–C are pathogenic to humans [1]. The HRV/EV genome is represented by a single-stranded positive-sense RNA, consisting of a 5’ untranslated region (UTR), an open reading frame (ORF) that encodes a single precursor polypeptide, and a 3’ untranslated region with a poly-A-tail. The precursor polypeptide undergoes post-translational processing, resulting in the formation of four structural proteins (VP1–4) and seven non–structural proteins. On the basis of amino acid sequence, divergence of the VP4/2 genes or VP1 genes EV and HRV are divided into more than 100 types [2]. Despite their genetic similarity, rhinoviruses and enteroviruses cause a broad spectrum of diseases [3]. Rhinoviruses are one of the main causes of acute respiratory tract infections [4]. Most often, HRV causes mild self-limited diseases but can also lead to severe lower respiratory tract illnesses such as bronchiolitis and pneumonia, as well as exacerbations of bronchial asthma or chronic obstructive pulmonary disease [5]. At the same time, EV can infect various tissues and can cause diseases of the gastrointestinal tract, respiratory, and nervous systems, such as hand-foot-and-mouth disease, viral meningitis, encephalitis, acute flaccid paralysis [6]. Some EV genotypes are predominantly isolated from respiratory samples, e.g., enteroviruses EV-C104, EV-C105, EV-C109, EV-C117, EV-C118, CV-A21, EV-D68 [3]. These genotypes cause respiratory tract diseases of varying severity and can, in some cases, lead to acute flaccid paralysis [7]. Particularly, EV-D 68 caused a major outbreak of severe respiratory disease and neurological complications in children in 2014, affecting more than 2000 people worldwide, primarily children [8]. Now, it continues to represent a global public health concern [9].

Since 2020, the COVID-19 pandemic and associated disease control measures have significantly altered the circulation patterns of enveloped viruses, including the influenza virus, respiratory syncytial virus (RSV), and human metapneumovirus. Concurrently, studies have shown that the circulation of rhinoviruses and enteroviruses has remained stable, continuing to be an important component of the respiratory viral spectrum [10]. However, little is known about the genetic diversity of enteroviruses and rhinoviruses in children with acute respiratory infections in Russia.

In this study, we assessed the contribution of enteroviruses to the etiology of acute respiratory infections in hospitalized children during the 2023–2024 period. We examined age distribution, clinical signs of illness, and the genetic diversity of human rhinovirus (HRV) and enteroviruses (EV). This research aims to fill a critical gap in understanding the epidemiological landscape of these viruses in pediatric populations within Russia.

## 2. Materials and Methods

### 2.1. Ethics Issues

The study was approved by the Committee on Biomedical Ethics at FRC FTM (protocol No. 16 of 15 June 2023). Each patient/legal representative involved in the study signed an informed consent.

### 2.2. Sample Collection

The study included 1992 children aged from 0 to 17 years hospitalized with acute respiratory infection (ARI) in two hospitals: Novosibirsk Children’s Municipal Clinical Hospital No. 6, which provides medical care to approximately 2000 patients with ARI, bronchitis, and pneumonia annually, and Novosibirsk Children’s Municipal Clinical Hospital No. 3, which provides medical care to 5000 patients with ARI, bronchitis, and pneumonia annually. The inclusion criteria were a duration of the disease of less than 7 days, the presence of at least one systemic symptom (fever, malaise, myalgia, headache), and one respiratory symptom (cough, sore throat, runny nose/nasal congestion, shortness of breath). On the first day after admission, a nasal and throat swab was taken from the child and placed in a tube with the transport medium (MEM medium, 0.5% bovine serum albumin, 100 mcg/mL gentamicin). The samples were stored at −20 °C before delivery to the laboratory, but no more than 72 h. In addition, a questionnaire was filled out for each patient, which reflected the main demographic data (gender, age), clinical symptoms of the disease, the presence of chronic diseases, hospitalization in the ICU, and the clinical diagnosis.

### 2.3. Detection of Respiratory Viruses

Total RNA/DNA was isolated from respiratory samples using a commercial Ribosorb kit (Interlabservice, Moscow, Russia) in accordance with the manufacturer’s instructions. Reverse transcription was performed using the Reverta-L kit (Interlabservice, Moscow, Russia) in accordance with the manufacturer’s instructions.

The detection of influenza (HInfV) and SARS-CoV-2 viruses was performed using the AmpliPrime Influenza SARS-CoV-2/Flu(A/B/H1pdm09) PCR kit (NextBio, Moscow, Russia). Respiratory syncytial virus (HRSV); coronaviruses NL63/229E, OC43/HKU1 (HCoV); parainfluenza virus types 1–4 (HPIV); metapneumovirus (HMPV); rhinovirus (HRV); adenovirus (HAdV); and bocavirus (HBoV) were detected using the AmpliSens ARVI-screen-FL PCR kit (Interlabservice, Moscow, Russia). The detection of enteroviruses was carried out using the AmpliSens Human enterovirus-FL PCR kit (Interlabservice, Moscow, Russia).

### 2.4. HRV/EV Typing

Nasal and throat swabs were suspended in 1000 µL of transport medium and centrifuged at 3000 rpm for 3 min. RNA extraction was carried out using the RNA Isolation Column Kit (modified) (Biolabmix, Novosibirsk, Russia) according to the manufacturer’s recommendations and stored at −80 °C until further analysis.

Sequencing of the VP1 capsid protein gene portion was performed in accordance with WHO recommendations [11]. Extracted RNA was then reverse-transcribed into cDNA using a reverse transcriptase RNAscribe RT kit (Biolabmix, Novosibirsk, Russia) and a set of primers (Table 1) for the VP1 gene (AN32, AN33, AN34, and AN35 in concentration 10 pmol/μL). Following incubation at 25 °C for 10 min, 55 °C for 50 min, and 85 °C for 5 min, the entire 20 μL RT reaction mixture was utilized for the first PCR (PCR1), which had a final volume of 50 μL. This reaction included primers SO224 and SO222 in a concentration 10 pmol/μL (Table) and was conducted using the BioMaster HS-Taq PCR-Color (2×) kit (Biolabmix, Novosibirsk, Russia) under the following conditions: 95 °C for 5 min, followed by 39 cycles of 95 °C for 20 s, 42 °C for 30 s, and a ramp of 0.4°C/s to 60 °C for 45 s, then 72 °C for 45 s, and a final extension at 72 °C for 5 min. Semi-nested PCR2 was conducted using 1 μL of the PCR1, primers AN88 and AN89 (10 pmol/μL) employing the BioMaster HS-Taq PCR-Color (2×) kit (Biolabmix, Novosibirsk, Russia) under the following conditions: at 95 °C for 5 min prior to 39 amplification cycles of 95 °C for 20 s, 60 °C for 20 s, and 72 °C for 15 s followed by a final incubation at 72 °C for 5 min. The result of PCR2 is a product of a VP1 gene fragment of 348–393 bp. The reaction products were separated and visualized on 1.5% agarose gels with ethidium bromide (0.5 μg/mL). The appropriate size products were purified from the gel using the GeneJET Gel Extraction Kit (Thermo Fisher Scientific, Waltham, MA, USA). The resulting DNA templates were sequenced with a BigDye Terminator v3.1 ready reaction cycle sequencing kit on an ABI 3500xL Genetic Analyzer (Applied Biosystems, Thermo Fisher Scientific, Foster City, CA, USA) by using primers AN89 and AN88. A total of 77 sequences of the partial VP1 203–353 nt were deposited in GenBank (accession numbers rhinoviruses: PQ539483-PQ539537, accession numbers enteroviruses: PQ519611-PQ519632). Sequences obtained were compared with the enterovirus sequences available in NCBI GenBank.

Finally, we selected samples for further sequencing from HRV/EV positive cases with a PCR cycle threshold (Ct) value ≤ 25, totaling 111 out of 220 positive samples. From these, we chose those that had a concentration of 3 ng/µL or higher after elution for sequencing and subsequent typing. This process enabled us to reliably type 86 viruses. Of these, 78 viruses were subjected to phylogenetic analysis, as their sequence quality and length met the necessary criteria for analysis.

### 2.5. Phylogenetic Analysis

Sequences of fragments of the VP1 region of enteroviruses and rhinoviruses obtained from this study were compared against nucleotide sequences from the relevant taxonomy group downloaded from NCBI GenBank. All sequences were then aligned using the MAFFT-L-INS-i (v7.520) [12]. Phylogenetic trees were constructed using the Maximum Likelihood (ML) method implemented in IQ-TREE IQtree v1.6.12 [13]. An ultrafast bootstrap (UFBoot) [14] and an approximate likelihood ratio test with the Shimodaira-Hasegawa-like (SH-aLRT) procedure [15] were used to evaluate support for nodes on the tree for 1000 replicates. Visualization was done with iTOL [16].

### 2.6. Statistical Analysis

Descriptive statistics were performed with median and interquartile range for quantitative variables, with absolute, relative frequencies and 95% confidence interval for categorical variables. The Manna-Whitney (U) test and the Chi-square test (Chi2) were applied to identify the statistical significance. Differences between groups were analyzed by the Kruskal-Wallis test (H) and multiple corrections were performed using Dunn’s post hoc test with Bonferroni correction. To quantify effect size, we used risk ratio (RR) with 95% CI, as well as the adjusted standardized residual (AR). An adjusted standardized residual greater than 1.96 indicates a statistically significant difference from the expected frequencies. The relationship between age and the presence of EV/HRV was assessed with Spearman’s rank correlation. The *p*-values were computed using permutation techniques with 999,999 iterations. The statistical significance was set to *p* < 0.05. The analysis was performed using R v.4.3.2, Python v.3.12, RStudio software, and GraphPad Prism v.10.3.1.

## 3. Results

The study was conducted from September 2023 to May 2024, coinciding with a seasonal increase in the incidence of acute respiratory infections. As part of the annual monitoring of respiratory viruses in hospitalized children, nasal and pharyngeal swabs were taken within 24 h of admission and subsequently analyzed by real-time PCR to detect common respiratory viruses. A total of 1992 samples were collected from children aged 0–17 years. Of these, 1098 (55.1%) samples were obtained from boys and 894 (44.9%) samples were obtained from girls. The largest number of samples was obtained from infants. The gender and age distribution of the patients is presented in Table 2.

### 3.1. Detection of Respiratory Viruses

**Figure 1:**
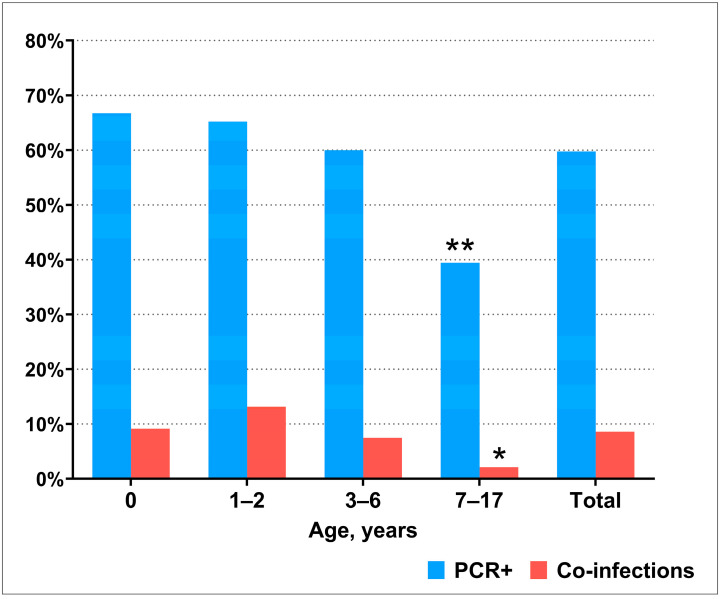
PCR detection rate of respiratory viruses in different age groups. Asterisks indicate differences between the 7–17–year age group and other age groups in the presence of respiratory viruses (**, AR = −8.96, *p* < 0.001) and co-infection (*, AR = −4.99, *p* < 0.001).

PCR testing revealed that at least one respiratory virus was detected in 1190/1992 (59.8%) of the included patients. Viral co-infection was detected in 171/1992 (8.6%) children. To identify age differences, we divided patients into the following age groups: children in the first year of life (0–12 months), young children (1–2 years), preschool children (3–6 years), and school-age children (7–17 years). The detection rate of respiratory viruses, as well as the frequency of viral co-infection, decreased with age being significantly lower in the age group of 7–17-year age group compared with all other ages (Figure 1).

**Figure 2:**
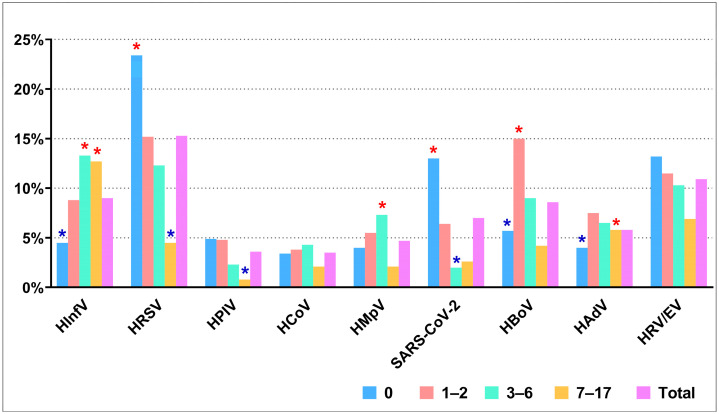
Age distribution of respiratory viruses in hospitalized children in 2023–2024. The prevalence of respiratory viruses significantly varies among age groups (Chi2 = 193.24, *p* < 0.001). Blue asterisks indicate negative AR, suggesting lower-than-expected frequencies, while red asterisks represent positive AR, indicating higher-than-expected frequencies, both with *p* < 0.001.

HRSV was the most prevalent in the etiology of ARIs during 2023–2024, which was detected in 305/1992 (15.3%) children. HRSV was significantly more frequently detected in children under 12 months of age compared with all other age groups (AR = 5.67, *p* < 0.001), with detection rates of 23.4%, 15.2%, 12.3%, and 4.5% for the respective age groups. A similar pattern was observed for SARS-CoV-2, which was significantly more common in children aged 0–12 months compared to children aged 1–2 years old, 3–6 years old, and 7–17 years old (AR = 6.41, *p* < 0.001) with detection rates of 13.0%, 6.4%, 2.0%, and 2.6%, respectively. HInfV was found in 179/1992 (9.0%) children, with the detection rate increasing with age; it was significantly higher in preschool (AR = 3.60, *p* < 0.001) and school-age (AR = 6.83, *p* < 0.001) children compared to those aged children 0–12 months and 1–2 years old with rates 13.3%, 12.7%, 4.5%, and 8.8%, respectively. The incidence of respiratory viruses across different age groups is shown in Figure 2.

### 3.2. Detection of HRV/EV

HRV detection was carried out using the ARVI-screen-FL PCR kit (Interlabservice, Moscow, Russia) and EV detection was performed using the AmpliSens Human enterovirus-FL PCR kit (Interlabservice, Moscow, Russia). The limitation of these kits includes potential cross-reactivity and the identification of closely related variants of enteroviruses and rhinoviruses, as noted in the manufacturer’s instructions. In our study, we found that out of 220 samples positive for HRV/EV by PCR, 121 samples were positive only for HRV, 49 samples were positive only for EV, and 50 samples detected both HRV and EV simultaneously.

There was no significant difference between sex and the presence of HRV/EV (*p* = 0.946). However, there was a statistically significant, negative, very weak correlation between age and the presence of HRV/EV (rs = −0.070, *p* = 0.002). In children under 1 year of age, the risk ratio for EV infection was 1.35 (95% CI: 1.05 to 1.74) (Table 3).

**Figure 3:**
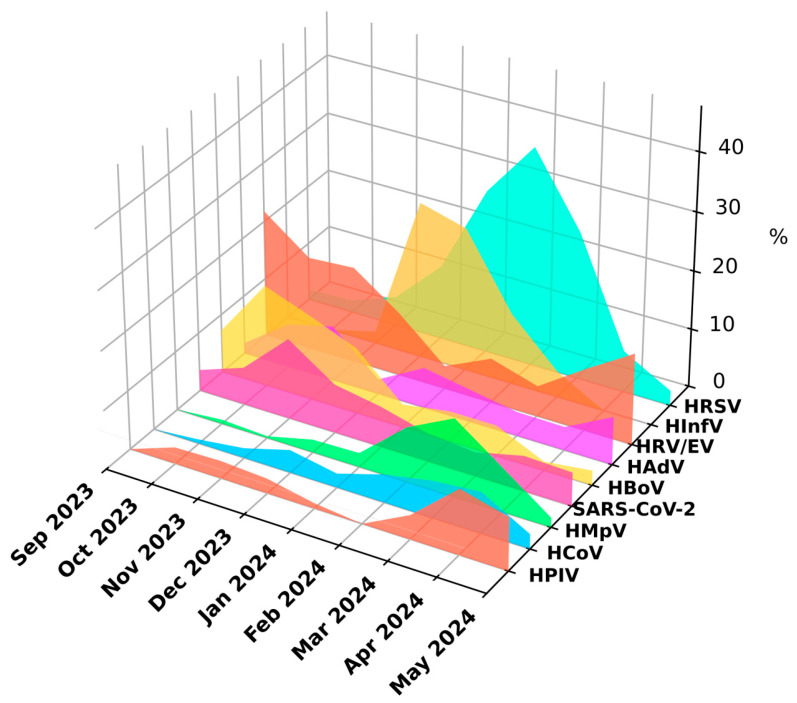
Seasonal distribution of respiratory viruses in 2023–2024.

**Figure 4:**
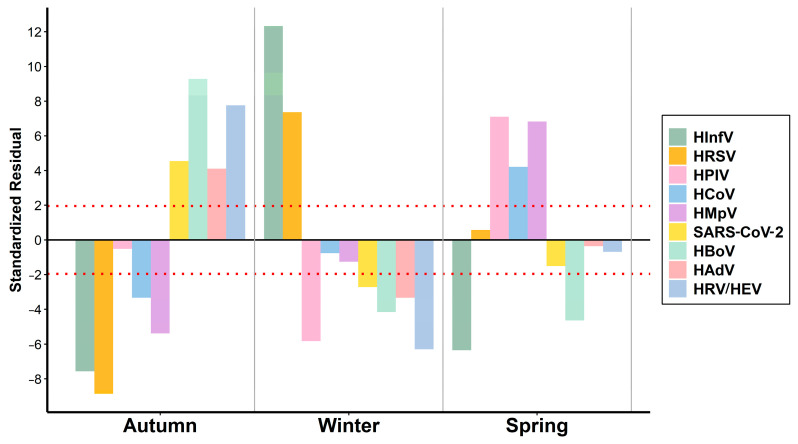
The bar chart shows the standardized residuals obtained from the chi-square test. These residuals serve as a measure of how much each category deviates from what would be anticipated under the null hypothesis, highlighting significant disparities in virus distribution across seasons. The red dotted line indicates the critical value of |1.96|, representing the 95% confidence threshold.

The prevalence of different respiratory viruses was not the same at different times of the year (Chi2 = 492.76, *p* < 0.001). HRV/EV were detected throughout the study period, with the peak infection rate in the autumn months (AR = 7.62, *p* < 0.001). During the winter months, there was a significant decrease in the detection levels of HRV/EV (AR = −6.30, *p* < 0.001) attributed to the prevalence of HInfV (AR = 12.34, *p* < 0.001) and HRSV (AR = 7.37, *p* < 0.001) (Figure 3 and Figure 4).

### 3.3. Clinical Signs of HRV/EV Infections

**Figure 5:**
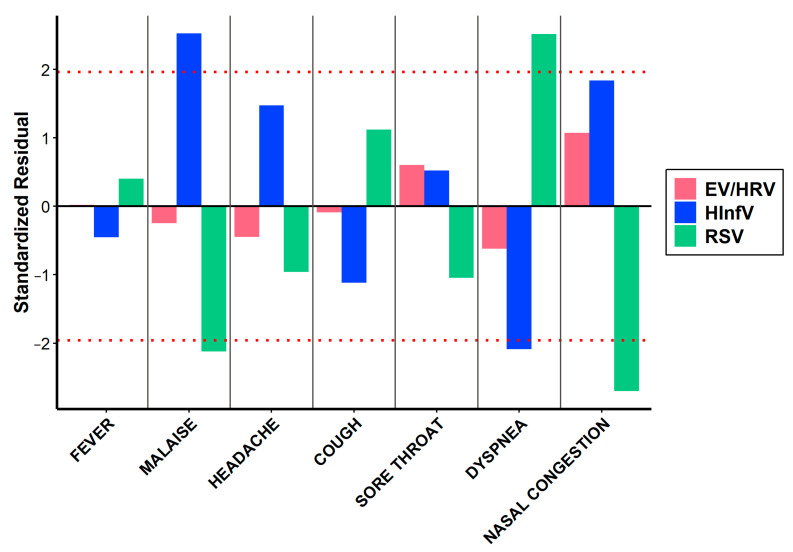
The bar chart shows the standardized residuals obtained from the chi-square test. These residuals help estimate how much the observed frequencies differ from the expected ones, which allows us to identify statistically significant deviations in the distribution of symptoms. The red dotted line indicates the threshold |1.96|, which corresponds to the 95% significance level.

We compared the clinical features of HRV/EV infection with those of Influenza and RSV infection. The distribution of various symptoms among children with respiratory viruses, HRV/EV, differs significantly (*p* = 0.020). However, the contribution to these differences is primarily due to the symptom distribution associated with HInfV and HRSV rather than HRV/EV (Table 4). Malaise was significantly more common in children with HInfV (AR = 2.52) and less frequent in those with HRSV (AR = −2.12) (Figure 5). Conversely, dyspnea was more frequently observed in children with HRSV (AR = 2.52) but less so in those with HInfV (AR = −2.09) (Figure 5). Nasal congestion was significantly less common in children with HRSV (AR = −2.69) (Figure 5). Statistically significant differences in temperature were found between HRV/EV, HInfV, and HRSV (*p* < 0.001). Post hoc comparisons revealed differences between all pairs: HRV/EV and HInfV (*p* < 0.001), HRV/EV and HRSV (*p* = 0.001), and HInfV and HRSV (*p* < 0.001). Statistically significant differences in the frequency of hypoxia were found between HRV/EV, HInfV, and HRSV (*p* = 0.015). Post hoc comparisons revealed differences between HRV/EV and HRSV (*p* = 0.030). No statistically significant differences in ICU admission were found (*p* = 0.177).

### 3.4. HRV/EV Typing and Phylogenetic Analysis

By partial sequencing of the VP1 gene, we performed typing of samples positive for HRV/EV. A total of 58 HRV and 28 EV samples were typed, including one rhinovirus sample and one enterovirus sample, which were collected during the 2022–2023 epidemic season. Rhinoviruses A (32 samples, 15 genotypes) were the most common among HRV, followed by rhinoviruses C (23 samples, eight genotypes). Rhinoviruses B were detected in only three cases (three genotypes). Enteroviruses were represented by all four species, with enterovirus D being the most frequently detected (13 samples), all belonging to the EV-D68 genotype. Enteroviruses A were represented by the genotypes coxsackievirus A2 and coxsackievirus A6. Among enteroviruses B, we identified coxsackievirus B 5, echovirus 5, and echovirus 6. Enterovirus C was represented by a single sample EV-C105 from the 2022–2023 season. The prevalence of HRV/EV genotypes is shown in Table 5.

**Figure 6:**
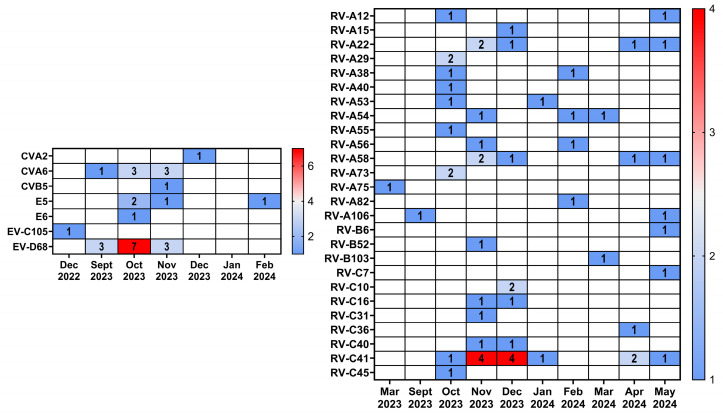
Heatmap showing the occurrence of EV or RV among different months.

A comparison of the seasonal distribution of HRV and EV revealed that rhinoviruses were detected throughout the entire observation period (September 2023–May 2024), while the vast majority of enteroviruses were detected in the fall of 2023 (Figure 6).

**Figure 7:**
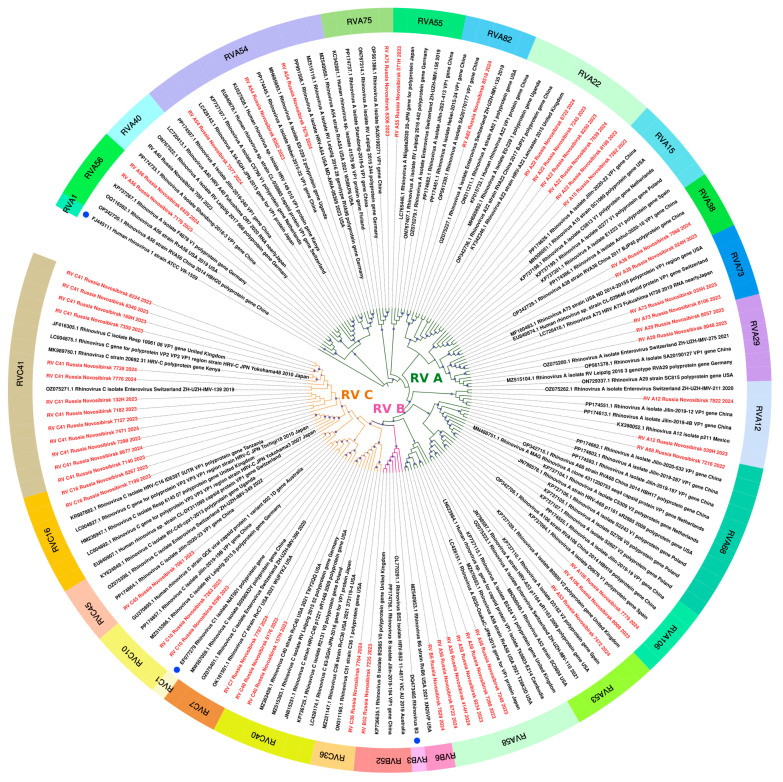
The phylogenetic tree constructed from partial VP1 nucleotide sequences of rhinoviruses (161 sequences). The analysis was inferred using the Maximum Likelihood method (substitution model: GTR + F + I + G4 + R). Reference strains for each species are shown with a blue circle; current study strains (n = 56) are shown in red. Colored bars indicate the genotype. Ultrafast bootstrap > 70% and SH-aLRT > 60% support values (n = 1000) are marked on the branches with purple circles, the size of which reflects the level of support.

The sequences of 56 rhinoviruses identified in this study belonged to three different species and exhibited similarities with various strains collected worldwide (Europe, East Asia, America, Africa, and Australia) between 2003 and 2023 (Figure 7). Given the extensive development of international air travel, tracking patterns of transmission has become a challenging task.

**Figure 8:**
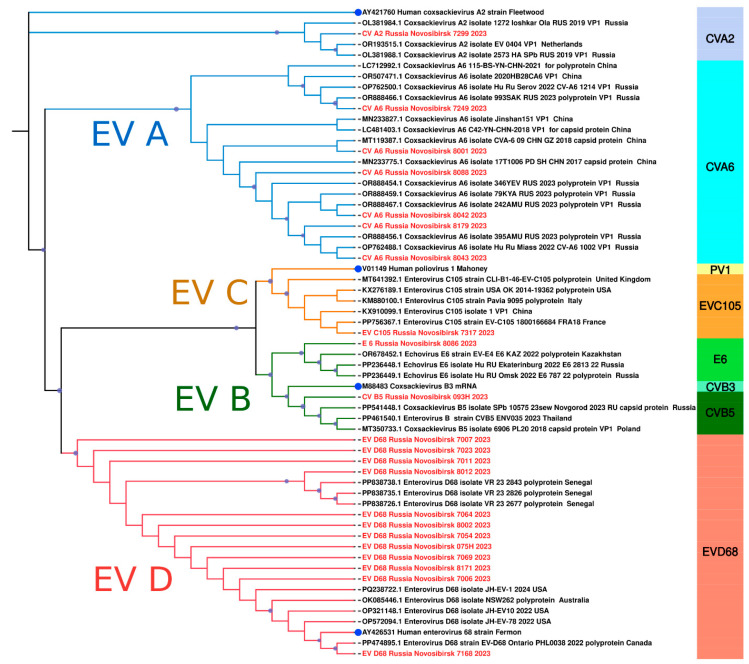
The phylogenetic tree constructed from partial VP1 nucleotide sequences of enteroviruses (61 sequences). The analysis was inferred using the Maximum Likelihood method (substitution model: K2P + G4 + R). Reference strains for each species are shown with a blue circle; current study strains (n = 22) are shown in red. Colored bars indicate the genotype. Ultrafast bootstrap > 70% and SH-aLRT > 60% support values (n = 1000) are marked on the branches with purple circles, the size of which reflects the level of support.

A detailed comparative analysis of the nucleotide sequences of Novosibirsk isolates coxsackievirus A6 showed an appreciable level of similarity with strains detected in 2017 and 2023 in various cities of Russia (Khabarovsk, Miass) as well as other countries, such as China and The Netherlands (Figure 8). Another genotype of EV, coxsackievirus A2, found in our study exhibited significant genetic similarity to a strain identified in the Russian city Yoshkar-Ola in 2019 (Figure 8), which was isolated from a throat swab. The Novosibirsk strain of coxsackievirus B5 demonstrated a close similarity of the nucleotide sequence of VP1 with the strain from Novgorod, which circulated in 2023 (Figure 8). Furthermore, we observed that the echovirus 6 strain had genetic similarities with those circulated in 2022. Two cases of echovirus 6 were detected in patients from Omsk and Yekaterinburg (Figure 8), with biological material sourced from cerebrospinal fluid and feces. Outside the Russian Federation, this strain was found in throat swabs collected in Kazakhstan (Figure 8).

It is especially important to emphasize that in our study, we detected a strain of EV-C105 for the first time in Russia, similar to the strain from France identified in a throat swab in 2018 (Figure 8).

**Figure 9:**
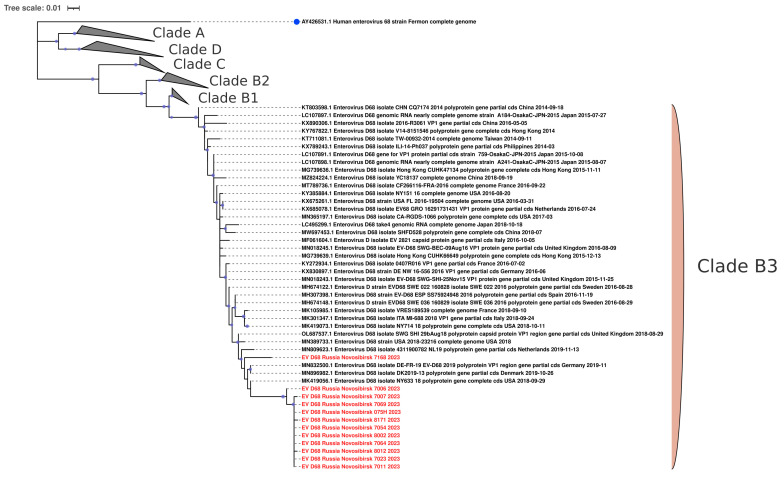
The phylogenetic tree constructed from partial VP1 nucleotide sequences of enteroviruses D68 (139 sequences). The tree includes GenBank sequences belonging to known clades. The analysis was inferred using the Maximum Likelihood method (substitution model: TN + F + G4 + R). Reference strain for enterovirus D68 is shown with a blue circle; current study strains (n = 12) are shown in red. Ultrafast bootstrap > 70% and SH-aLRT > 60% support values (n = 1000) are marked on the branches with purple circles, the size of which reflects the level of support.

Additionally, our investigation revealed several sequences of EV-D68, the vast majority of which exhibited a high degree of genetic similarity to those collected from the nasopharyngeal swabs of children in Senegal (Africa) in 2023 (Figure 8). Interestingly, one strain was closely related to a variant from Canada, sourced from a nasopharyngeal swab in 2022 (Figure 8). Phylogenetic analysis of the VP1 fragment showed that all our sequences belonged to the B3 subclade of enterovirus D68 (Figure 9).

### 3.5. Clinical Characteristics of HRV-A and HRV-C Infection

To examine previous reports on the differences in clinical characteristics (particularly severity) between HRV subtypes, we compared the clinical characteristics of diseases caused by HRV-A and HRV-C in order to identify the possible effect of the virus type on the disease severity. No statistically significant differences were found; however, a tendency toward statistically significant differences between HRV-A and HRV-C was found in age (*p* = 0.090), presence of hypoxia (*p* = 0.050), and diagnoses (*p* = 0.076, due to the low proportion of upper respiratory tract diseases in children with HRV-C) (Table 6).

### 3.6. Clinical Characteristics of EV-D68 Infection

To verify the differences between our study and previous reports that EV-D68 causes neurological manifestations in addition to respiratory disease, we have analyzed the clinical characteristics of EV-D68 infection. In total, we have confirmed 13 cases of EV-D68 infection. Most of the patients were young children with a median age of 18 months. All children had no chronic diseases. All patients had a fever (median Temperature was 38.3 °C), 76.92% of patients had a cough, and 69.23% had shortness of breath. Almost half of the cases occurred with hypoxia, but none of the patients needed oxygen support, mechanical ventilation, or hospitalization in the ICU. All patients with EV-D68 had low respiratory tract infections, including acute bronchitis in 61.54% of cases, and pneumonia in 38.46% of cases. None of the patients exhibited neurological symptoms (Table 7).

## 4. Discussion

Rhinoviruses and respiratory enteroviruses remain one of the leading causes of acute respiratory infections, especially in children [3,17]. In our study, we found that the HRV/EV ratio reached 18.5% (220/1190) among PCR-confirmed viral infections in hospitalized children in 2023–2024 in Novosibirsk. Rhinoviruses are considered the primary cause of the so-called “common cold” with mild symptoms, but they can cause severe diseases of the lower respiratory tract, requiring hospitalization, such as bronchitis, bronchiolitis, and pneumonia [5,18]. In our study, 53.6% (118/220) of children with RV/EV had bronchitis and 29.5% (65/220) of the children had pneumonia.

Rhinoviruses and enteroviruses are heterogeneous groups of viruses, including over 169 RV genotypes of HRV and 116 Genotypes of EV, according to the International Committee on Taxonomy of Viruses (ICTV) [19]. We detected 32 viruses of HRV-A, which belonged to 15 different genotypes, 23 HRV-C, which belonged to eight genotypes, and only three HRV-B, which belonged to three genotypes. According to different studies, RV-A and RV-C are also predominant in the world [20,21,22]. In the review of Esneau C. [5], based on the analysis of studies conducted in different regions, the top 25 subtypes of rhinoviruses in Europe, Asia, and Africa were identified. In our study, RV-C41 was the most often detected (13/58), which, however, was not in the 25 highest-ranked subtypes in the Esneau C. study [5].

HRV infections occur year-round; however, seasonality is characterized by a major peak of infection in the autumn/fall and a smaller one in the spring [23], while EV infection peaks in summer and early autumn [24]. We also could see HRV infection throughout the year, with two peaks in the autumn and spring months and the majority of cases of confirmed EV infection occurring in the fall of 2023.

Some investigators have reported that RV-C caused more serious illness compared with cases caused by RV-A and RV-B [23,25]. However, other studies have not found differences in the disease severity [21,26]. Some researchers note that low respiratory tract infection was more common in HRV-C than HRV-A illness cases [25]. We did not find a statistically significant difference in the severity of the diseases due to the small number of analyzed cases, however we observed a tendency towards statistically significant differences in the presence of hypoxia and a lower proportion of upper respiratory tract diseases in children with HRV-C compared with HRV-A.

The most prevalent genotype of EV in our study was EV-D68. We detected 13 cases of EV-D68 infection, mostly in young children. All patients had low respiratory tract infection (bronchitis or pneumonia). In addition to respiratory tract infections, EV-D68 illnesses can be associated with neurologic diseases, such as acute flaccid myelitis [9]. None of the patients showed neurological symptoms in our study. Phylogenetic analysis of the VP1 fragment showed that all our sequences belonged to the B3 subclade of enterovirus D68, which is most often responsible for outbreaks of respiratory diseases caused by enteroviruses [27,28,29]. Intriguingly, the sequences of enterovirus D68 from Novosibirsk, Russia, are very similar to sequences of enteroviruses from different parts of the world, such as Africa and Canada. Such information indicates the possibility of transmission of enterovirus D68 between countries located at great distances. Moreover, homology is observed between strains found in one year in distant parts of the world (Novosibirsk, 2024 and Senegal, 2023). This indicates the high speed with which enterovirus D68 is able to cross continental boundaries.

In our study, we identified the first case of the EV-C105 infection in Russia in an HIV-positive two-year-old girl hospitalized with pneumonia, and it was detected in combination with Coronavirus HKU-1/OC 43. The girl had only respiratory symptoms and no neurological manifestations. Phylogenetically, the Novosibirsk strain EV-C105 was close to the strain discovered in France in 2018. The genotype C105 was discovered less than two decades ago, and there are currently only 34 sequences in NCBI GenBank. It was first identified in the Democratic Republic of the Congo in 2010 from patients with acute flaccid paralysis [30]. Thus, it can be inferred that either 14 years or less have passed before the virus transferred from Africa to Europe and then to the Asian part of Russia. Nevertheless, this is a hypothesis as there are not many sequenced strains of enteroviruses, especially for this genotype, as mentioned earlier.

Phylogenetic analysis of Coxsackievirus A6 strains showed their similarity with strains from different cities of Russia (Khabarovsk, Miass) as well as other countries (China and The Netherlands). The wide geographic distribution of Coxsackievirus A6 viruses indicates the strain can travel long distances. At the same time, between 2020 and 2022, many countries introduced quarantine measures that reduced the volume of passenger and cargo shipments, which may have slowed but did not stop the spread of the virus.

The observed similarities between the detected enterovirus strains (coxsackieviruses, echovirus) in Russia may indicate related sources of infection and the ability of the strains to circulate in the population for several years. In addition, the genetic similarity of most strains to those detected in countries such as China, The Netherlands, and Kazakhstan indicates a broader circulation pattern that may include international transmission routes.

The implementation of non-pharmaceutical interventions aimed at mitigating the spread of SARS-CoV-2 led to a marked decline in the activity of many respiratory viruses. For instance, global influenza virus activity saw a substantial reduction during the early months of the pandemic, as non-pharmaceutical interventions effectively curtailed transmission routes shared with other respiratory viruses [31]. RSV also experienced significant decreases in case counts across various regions, particularly affecting pediatric populations where close contact in settings like childcare facilities is common. In contrast, rhinoviruses and enteroviruses demonstrated resilience during this period. While their prevalence initially declined at the pandemic’s onset, they quickly rebounded and maintained levels comparable to those observed in previous years [32]. This resilience may be attributed to their transmission dynamics and environmental stability, which allowed them to persist even amidst stringent public health measures. Overall, the COVID-19 pandemic has reshaped our understanding of respiratory virus epidemiology. The reductions in circulation for many enveloped viruses highlight the effectiveness of control measures, while the sustained presence of rhinoviruses underscores their unique characteristics within the respiratory viral landscape. Continued surveillance and research are essential to inform future public health strategies as we navigate the ongoing impacts of these changes.

## Figures and Tables

**Table 1:** Primers used for cDNA synthesis and PCR amplification (according to WHO recommendations) [11].

Step	Primer Name	Sequence
cDNA (RT) primers	AN32	5′ GTY TGC CA 3′
	AN33	5′ GAY TGC CA 3′
	AN34	5′ CCR TCR TA 3′
	AN35	5′ RCT YTG CCA 3′
PCR 1 primers	SO224 (forward)	5′ GCI ATG YTI GGI ACI CAY RT 3′
	SO222 (reverse)	5′ C ICC IGG IGG IAY RWA CAT 3′
snPCR 2 primers	AN89 (forward)	5′ CCA GCA CTG ACA GCA GYN GAR AYN GG 3′
	AN88 (reverse)	5′ TAC TGG ACC ACC TGG NGG NAY RWA CAT 3′

**Table 2:** Demographic characteristics of included patients.

Age	Sex		
	Male	Female	Total
0–12 months	372 (18.7%)	295 (14.8%)	667 (33.5%)
1–2 years	318 (15.9%)	229 (11.5%)	547 (27.4%)
3–6 years	213 (10.7%)	187 (9.4%)	400 (20.1%)
7–17 years	195 (9.8%)	183 (9.2%)	378 (19.0%)
Total	1098 (55.1%)	894 (44.9%)	1992 (100%)

Note: the percentage is calculated from the total number of collected samples.

**Table 3:** Distribution of HRV/EV among different age groups. Absolute, relative frequencies with 95% CI and relative risk of HRV/EV infection are indicated.

Age	Presence of EV/HRV	Risk Ratio of EV/HRV Infection, 95%CI
0 years (n = 667)	89, 13.34% [10.97–16.14]	RR = 1.35 [1.05–1.74] *
1–2 years (n = 547)	64, 11.70% [9.27–14.67]	RR = 1.08 [0.82–1.43]
3–6 years (n = 400)	41, 10.25% [7.65–13.61]	RR = 0.91 [0.66–1.26]
7–17 years (n = 378)	26, 6.88% [4.74–9.89]	RR = 0.57 [0.39–0.85] *

*—indicates statistical significance.

**Table 4:** Distribution of symptoms among the most common respiratory viruses and enteroviruses/rhinoviruses. Absolute, relative frequencies, and 95% CI are indicated.

Symptoms	HRV/EV (n = 220)	HInfV (n = 178)	RSV (n = 306)	Comparison
Fever	161, 73.18% [66.81–78.91]	161, 90.45% [85.15–94.34]	231, 75.49% [70.27–80.21]	Chi^2^ = 23.75; *p* = 0.020 *
Malaise	61, 27.73% [21.92–34.14]	80, 44.94% [37.49–52.56]	74, 24.18% [19.49–29.38]	
Headache	1, 0.45% [0.12–0.25]	3, 1.69% [0.35–4.85]	1, 0.33% [0–1.81]	
Cough	156, 70.91% [64.43–76.82]	151, 84.83% [78.70–89.76]	232, 75.82% [70.62–80.51]	
Sore throat	8, 3.64% [1.58–7.04]	8, 4.49% [1.96–8.66]	7, 2.29% [0.92–4.66]	
Dyspnea	70, 31.82% [25.72–38.42]	62, 34.83% [27.86–42.32]	123, 40.20% [34.66–45.93]	
Nasal congestion	45, 20.45% [15.33–26.40]	50, 28.09% [21.62–35.30]	41, 13.40% [9.79–17.73]	
Median Temperature, °C	38.3 (37.8–38.9)	39.4 (39.0–39.8)	38.6 (38.0–39.1)	H = 137.9, *p* < 0.001 *
Hypoxia	70, 31.82% [26.02–38.24]	58, 32.58% [26.13–39.77]	131, 42.81% [37.39–48.41]	H = 8.5, *p* = 0.015 *
ICU	7, 3.18% [1.55–6.42]	8, 4.49% [2.30–8.62]	5, 1.63% [0.70–3.77]	H = 3.5, *p* = 0.177

*—indicates statistical significance.

**Table 5:** HRV/EV genotypes distribution.

Species	Genotypes	Number of Samples
Rhinovirus A	RV-A12	2
	RV-A15	1
	RV-A22	5
	RV-A29	2
	RV-A38	2
	RV-A40	1
	RV-A53	2
	RV-A54	3
	RV-A55	1
	RV-A56	2
	RV-A58	5
	RV-A73	2
	RV-A75	1
	RV-A82	1
	RV-A106	2
Rhinovirus B	RV-B6	1
	RV-B52	1
	RV-B103	1
Rhinovirus C	RV-C7	1
	RV-C10	2
	RV-C16	2
	RV-C31	1
	RV-C36	1
	RV-C40	2
	RV-C41	13
	RV-C45	1
Enterovirus A	CV A2	1
	CV A6	7
Enterovirus B	CV B5	1
	E5	4
	E6	1
Enterovirus C	C105	1
Enterovirus D	D68	13
7 species	33 genotypes	86 viruses

**Table 6:** Distribution of the characteristics of children under study with HRV A and HRV C. Absolute and relative frequencies with 95% CI are indicated for qualitative variables, and medians with interquartile range are indicated for quantitative variables.

Characteristics	HRV A (n = 32)	HRV C (n = 23)	Comparison
Sex:			U = 348.5; *p* = 0.789
Female (n = 27)	17, 53.13% [36.45–69.13]	11, 47.83% [29.24–67.04]	
Male (n = 28)	15, 46.88% [30.87–63.55]	12, 52.17% [32.96–70.76]	
Median age, years	1 (0–3)	2 (1–5)	U = 270.5; *p* = 0.090
Symptoms:			Chi^2^ = 3.45; *p* = 0.644
Fever	31, 96.88% [83.78–99.92]	22, 95.65% [78.05–99.89]	
Malaise	14, 43.75% [26.36–62.34]	15, 65.22% [42.73–83.62]	
Headache	0, 0% [0–10.9]	0, 0% [0–14.8]	
Cough	32, 100% [89.11–100]	21, 91.30% [71.96–98.93]	
Sore throat	1, 3.13% [0.08–16.22]	3, 13.04% [2.78–33.59]	
Dyspnea	14, 43.75% [26.36–62.34]	15, 65.22% [42.73–83.62]	
Nasal congestion	7, 21.88% [9.28–39.97]	5, 21.74% [7.46–43.70]	
Median Temperature, °C	38.1 (37.4–38.5)	38.0 (37.4–38.4)	U = 368.0; *p* > 0.999
Hypoxia	8, 25.00% [13.25–42.11]	12, 52.17% [32.96–70.76]	U = 268.0, *p* = 0.050
Diagnosis:			Chi^2^ = 5,27; *p* = 0.076
Upper respiratory tract diseases	9, 28.13% [15.57–45.38]	1, 4.35% [0.22–20.99]	
Bronchitis	16, 50.00% [33.63–66.37]	14, 60.87% [40.79–77.84]	
Pneumonia	7, 21.88% [11.02–38.76]	8, 34.78% [18.81–55.11]	
ICU	2, 6.25% [1.11–20.15]	1, 4.35% [0.22–20.99]	U = 361.0, *p* > 0.999

**Table 7:** Clinical characteristics of EV-D68 infection.

Characteristics	EV-D68 (n = 13)
Median age, months	18 (1–96)
Symptoms:	
Fever	13, 100% [75.29–100]
Malaise	9, 69.23% [38.57–90.91]
Headache	1, 7.69% [0.19–36.03]
Cough	10, 76.92% [46.19–94.96]
Sore throat	2, 15.38% [1.92–45.45]
Dyspnea	9, 69.23% [38.57–90.91]
Nasal congestion	2, 15.38% [1.92–45.45]
Neurological symptoms	0, 0% [0–24.71]
Median Temperature, °C	38.3 (37.0–39.4)
Hypoxia	6, 46.15% [19.22–74.87]
ICU	0, 0% [0–24.71]
Diagnosis:	
Upper respiratory tract diseases	0, 0% [0–24.71]
Bronchitis	8, 61.54% [31.58–86.14]
Pneumonia	5, 38.46% [13.86–68.42]

## Data Availability

The original contributions presented in the study are included in the article; further inquiries can be directed to the corresponding author. Also, the nucleotide sequences of partial VP1 have been deposited in the NCBI GenBank database (rhinoviruses: PQ539483-PQ539537, enteroviruses: PQ519611-PQ519632).
